# Separation of Antioxidants from Trace Fraction of *Ribes himalense* via Chromatographic Strategy and Their Antioxidant Activity Supported with Molecular Simulations

**DOI:** 10.3390/ijms25010227

**Published:** 2023-12-22

**Authors:** Youyi Liu, Chuang Liu, Yuqing Lei, Jingrou Guo, Xingyi Chen, Minchen Wu

**Affiliations:** 1 Wuxi School of Medicine, Jiangnan University, Wuxi 214122, China; youyi@jiangnan.edu.cn (Y.L.); 7210201054@stu.jiangnan.edu.cn (C.L.); 7210201010@stu.jiangnan.edu.cn (Y.L.); 6212805002@stu.jiangnan.edu.cn (J.G.); 6212805001@stu.jiangnan.edu.cn (X.C.); 2 School of Biotechnology, Jiangnan University, Wuxi 214122, China

**Keywords:** antioxidants, *Ribes himalense*, chromatographic strategy, molecular docking

## Abstract

Antioxidants from natural sources have long been of interest to researchers. In this paper, taking the traditional Tibetan medicine *Ribes himalense* as an example, an integrated approach was used to identify and isolate its chemical composition with free-radical-scavenging properties from its ethanol extract. First, the ethanol extract of *Ribes himalense* was pretreated using polyamide medium-pressure liquid chromatography (polyamide-MPLC), and the target fraction (Fr4) was obtained. Then, a combined HPLC mode was utilized to purify antioxidants in Fr4 under the guidance of an online HPLC-1,1-diphenyl-2-picrylhydrazyl (HPLC-DPPH) activity screening system. Finally, three antioxidants (3-caffeoylquinic acid methyl ester, rutin, and myricetin-3′-α-L-rhamnopyranoside) were isolated, and this is the first report of their presence in *R. himalense*. Further molecular docking studies showed that the antioxidants exhibited good binding with HO-1, Nrf2, and iNOS. In conclusion, this comprehensive approach is capable of extracting high-purity antioxidants from trace fractions of *Ribes himalense* and holds promise for future applications in the exploration of the chemical compositions and bioactivity of natural products.

## 1. Introduction

In the process of development spanning several millennia in traditional Chinese medicine, Tibetan medicine has become one of the irreplaceable drug resources [1]. The unique geographical environment contributes to the abundance of secondary metabolites in Tibetan medicinal herbs, which also implies potential biological activities [2]. *Ribes himalense* Royle ex Decne (*R. himalense*), a small shrub of the Grossulariaceae family’s genus *Ribes*, and its free-radical-scavenging, detoxifying, anti-inflammatory, and vascular-disease-treating characteristics have been widely documented and inherited in classic Tibetan medicine [3,4,5]. In our previous study, a total of four fractions were recovered from the pretreatment of *R. himalense* extract using polyamide-MPLC, and the analysis of Fr2 and Fr3, which were the largest in amount, revealed that they were rich in flavonoids [6]. In order to further clarify the material basis of the medicinal effects of this plant and to advance its application in the modern medical system, it is imperative to carry out a systematic and scientific exploration of other fractions in *R. himalense*.

Considering the intrinsic properties of antioxidants and their affirmative impact on the treatment of several diseases, the development of natural and efficient exogenous antioxidants has been widely promising [7,8]. Consequently, the accurate identification of antioxidants within natural products stands as the foremost challenge to address. At the chromatographic level, an online HPLC-DPPH activity screening system based on the concept of post-column derivatization has been favored by scientists due to its high efficiency, targeting, and online advantages [9,10]. In this system, two HPLCs are connected via a mixer and a reaction coil. The sample flows through the columns and is mixed and reacted with the DPPH solution. The potential of compounds to scavenge free radicals is directly proportional to the decrease at 517 nm [11]. With this system, the online identification of free radical inhibitors in various natural products can be realized.

The development of HPLC has provided more options for the separation of abundant secondary metabolites in natural products [12]. In order to avoid column contamination and remove non-target components, crude samples need to be pretreated using medium-pressure liquid chromatography (MPLC) before HPLC separation. MPLC is a powerful isolated tool that has been used in natural product pretreatment, which can efficiently remove non-target components (biomacromolecules, fat-soluble components, etc.) and enrich trace ingredients [13,14]. The combined use of MPLC and HPLC can undoubtedly achieve the targeted, large-scale, and visual separation of compounds in natural products.

Conducting animal experiments directly on isolated compounds is likely to involve a loss of activity and resource inefficiency. Hence, it appears more prudent to initiate theoretical simulation studies as a preliminary step. Molecular docking currently represents a widely utilized computational technique in the field of drug discovery [15,16]. In the molecular docking of a compound (ligand) with a target protein of interest (receptor), the spatial conformation of the ligand is continuously altered to calculate the optimal binding mode and docking score with the receptor. Undoubtedly, these computational simulation techniques are providing robust assistance from a theoretical perspective in the study of the biological activity of compounds.

Considering the aforementioned reports, the chromatographic method was utilized to identify and isolate free radical inhibitors and then further explore their biological activities from a computerized perspective. Such an integrated strategy is expected to elucidate the material basis of their medicinal effects while providing a theoretical basis for the high-value processing of *R. himalense*.

## 2. Results

### 2.1. Polyamide-MPLC Enrichment and Antioxidant Recognition

**Figure 1:**
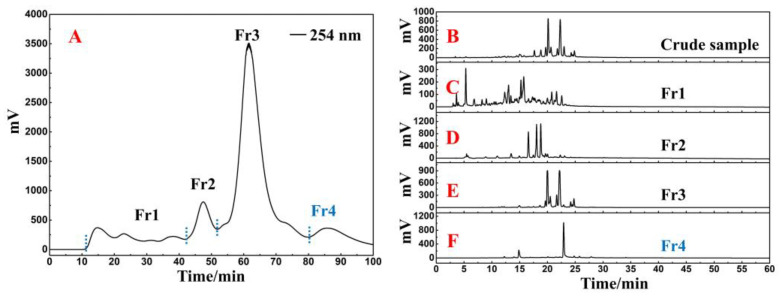
*R. himalense* extract–polyamide mixture separation chromatogram (**A**). The HPLC analysis of *R. himalense* crude sample (**B**) and Fr1–Fr4 (**C**–**F**) in ReproSil-Pur C18 AQ analytical column.

The crude sample was added to a small medium-pressure column (49 × 100 mm) connected to a polyamide medium-pressure column. After the pretreatment procedure was repeated 13 times, the recovered liquids were combined to finally obtain four fractions, of which the target fraction Fr4 weighed 1.4 g (the lightest weight of the four fractions). Figure 1A shows the separation chromatogram. The actual polyamide-MPLC setup diagram is shown in Figure S1A.

**Figure 2:**
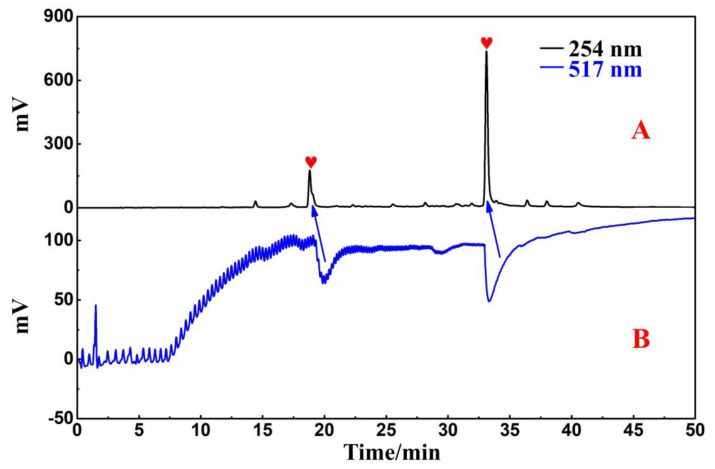
Analytical chromatogram (**A**) in Reprosil-Pur C18 AQ analytical column and free radical inhibitory activity profile (**B**) of Fr4.

In a Reprosil-Pur C18 AQ analytical column, the crude sample and four collected fractions were analyzed simultaneously. The analytical diagram (Figure 1B–F) shows that the main ingredients of *R. himalense* were effectively enriched in Fr2 and Fr3, while trace ingredients were mostly enriched in Fr1 and Fr4. Subsequently, the antioxidants in Fr4 were detected with the online HPLC-DPPH activity screening system. Figure S1B shows a schematic diagram of this system. As illustrated in Figure 2A,B, the retention time between 15 and 35 min displayed two significant negative peaks at 517 nm, thereby implying that the two peaks at 254 nm had free radical scavenging activity (marked with red shapes). These two active peaks had to be further purified and separated to obtain high-purity free radical inhibitors.

### 2.2. Directed Separation of Antioxidants from Fr4

**Figure 3:**
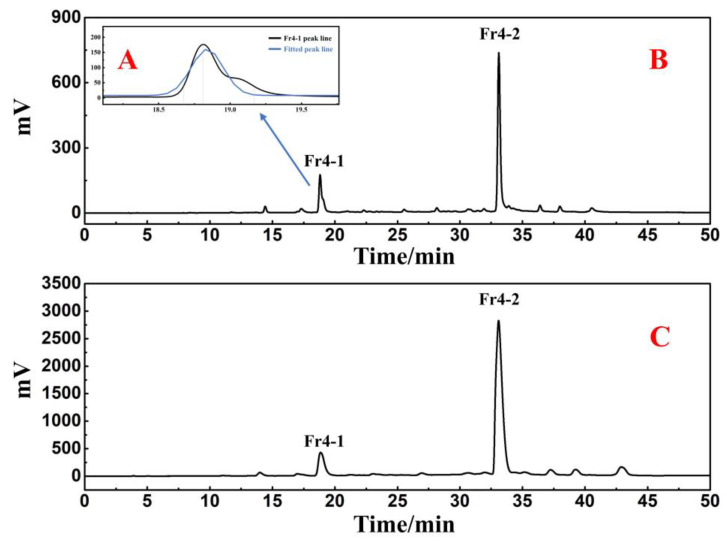
Partial enlargement of Fr4-1 (**A**). Comparison chromatogram of analysis (**B**) and preparation (**C**) of Fr4.

Fr4 was separated in a Reprosil-Pur C18 AQ preparative column using an injection volume of 0.5 mL and a flow rate of 19 mL/min. The analytical chromatogram and preparative chromatogram results of Fr4 are shown in Figure 3B,C. After repeating the purification method six times, two active fractions were finally collected and labeled as Fr4-1 (104.2 mg) and Fr4-2 (377.2 mg). The remaining inactive chromatographic peaks were directly discarded.

**Figure 4:**
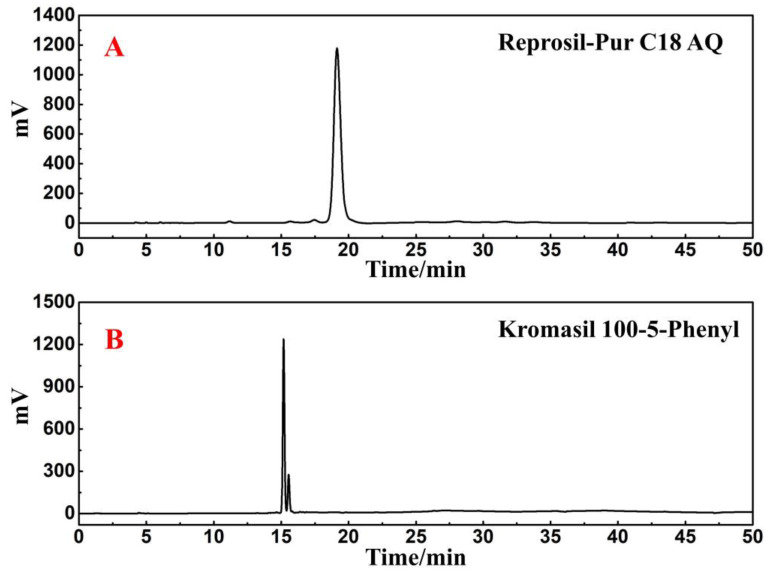
Analysis chromatogram of Fr4-1 in Reprosil-Pur AQ analytical column (**A**) and Kromasil 100-5-Phenyl analytical column (**B**).

**Figure 5:**
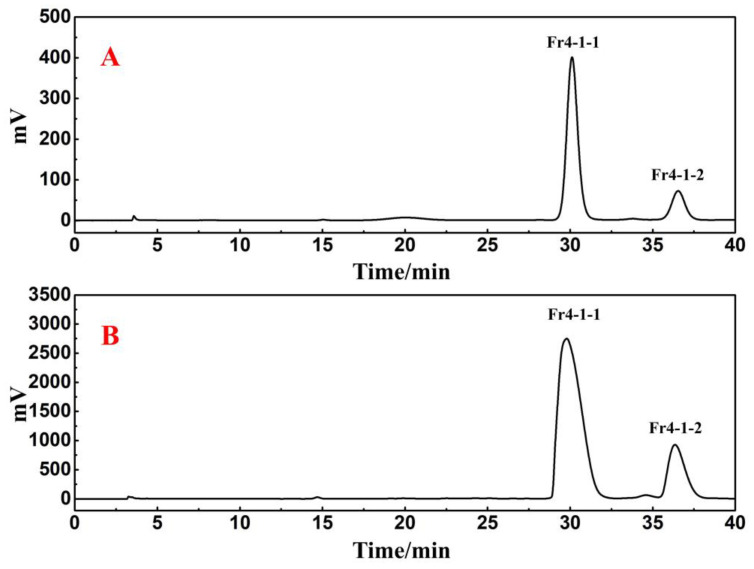
The optimized analytical chromatogram of Fr4-1 in Kromasil 100-5-Phenyl analytical column (**A**) and preparative chromatogram in Kromasil 100-5-Phenyl preparative column (**B**).

For Fr4-1, 104.2 mg was dissolved in 1.0 mL of methanol, and the sample solution was passed through a 0.45 μm filter preceding the study. Figure 4B shows two visible separated chromatographic peaks of Fr4-1 in the Kromasil 100-5-phenyl analytical column. However, in the case of the Reprosil Pur C18 AQ analytical column, the results still show a main chromatographic peak (Figure 4A). Based on the above findings, Fr4-1 was further separated using a Kromasil 100-5-phenyl preparative column. After further optimizing the conditions for the chromatographic separation of Fr4-1, two fractions labeled Fr4-1-1 (43.4 mg) and Fr4-1-2 (19.5 mg) were obtained via 10 preparative separation cycles. Figure 5 shows the analytical and preparative comparison chromatograms of Fr4-1. It can be seen that the retention times of these two target peaks in the preparation diagram (Figure 5B) of Fr4-1 were basically the same as that in the analysis diagram (Figure 5A), which did not affect the recovery of high-purity antioxidants.

### 2.3. Purity, Structural Characterization, and Activity of Isolated Antioxidants

Using online HPLC-DPPH analysis, the purity and activity of Fr4-1-1, Fr4-1-2, and Fr4-2 were all above 95%. The MS and NMR were acquired to elucidate the structures of the isolated antioxidants and compared with the data reported in the literature. The spectra obtained in this study are shown in the Supporting Information (Figures S3–S12). This is the first report of these three antioxidants in *R. himalense*.

Fr4-1-1: 3-caffeoylquinic acid methyl ester, light yellow powder, 43.3 mg, ESI-MS *m*/*z* 367.23, [M-H]^−^, calc. for C_17_H_20_O_9_, *m*/*z* 368.11. ^1^H NMR (600 MHz, DMSO-*d*_6_): 7.39 (1H, d, *J* = 15.9 Hz, H-7′), 7.02 (1H, d, *J* = 2.0 Hz, H-2′), 6.97 (1H, dd, *J* = 8.2, 2.0 Hz, H-6′), 6.77 (1H, d, *J* = 8.2 Hz, H-5′), 6.10 (1H, d, *J* = 15.9 Hz, H-8′), 5.01 (1H, dd, *J* = 9.1, 5.6 Hz, H-3), 3.87 (1H, m, H-4), 3.57 (1H, m, H-5), 3.55 (3H, s, H-7-CH_3_), 2.10 (2H, m, H-2a, 6a), 1.92 (1H, dd, *J* =13.6, 3.2 Hz, H-6b), and 1.76 (1H, dd, *J* = 12.5, 9.6 Hz, H-2b). ^13^C NMR(151 MHz, DMSO-*d_6_*): 173.6 (C-7), 165.4 (C-9′), 148.5 (C-4′), 145.6 (C-7′), 145.1 (C-3′), 125.4 (C-1′), 121.3 (C-6′), 115.8 (C-5′), 114.6 (C-2′), 113.8 (C-8′), 73.0 (C-1), 71.0 (C-5), 69.3 (C-4), 66.8 (C-3), 51.8 (7-O-CH_3_), and 37.3 (C-2), 35.0 (C-6). These data are consistent with the published data for 3-caffeoylquinic acid methyl ester [17,18].
Fr4-1-2: rutin, yellow powder, 19.5 mg, HR-ESI-MS *m*/*z* 611.1633 [M+H]^+^, calc. for C_27_H_30_O_16_, *m*/*z* 610.1534. ^1^H NMR (600 MHz, MeOH-*d*_4_): 7.66 (1H, d, *J* = 2.1 Hz, H-2′), 7.62 (1H, dd, *J* = 8.4, 2.1 Hz, H-6′), 6.87 (1H, d, *J* = 8.4 Hz, H-5′), 6.39 (1H, d, *J* = 2.0 Hz, H-8), 6.20 (1H, d, *J* = 2.0 Hz, H-6), 5.10 (1H, d, *J* = 7.7 Hz, H-1″), 4.51 (1H, d, *J* = 1.5 Hz, H-1‴), 3.80~3.25 (10H, m, sugar protons), and 1.11 (3H, d, *J* = 6.2 Hz, H-6‴). ^13^C NMR (151 MHz, MeOH-*d*_4_): 179.4 (C-4), 166.0 (C-7), 163.0 (C-5), 159.3 (C-9), 158.5 (C-2), 149.8 (C-4′), 145.8 (C-3′), 135.6 (C-3), 123.6 (C-1′), 123.1 (C-6′), 117.5 (C-5′), 116.0 (C-2′), 105.6 (C-10), 104.7 (C-1″), 102.4 (C-1‴), 99.9 (C-6), 94.9 (C-8), 78.2 (C-5″), 77.2 (C-3″), 75.7 (C-2″), 73.9 (C-4‴), 72.2 (C-3‴), 72.1 (C-2‴), 71.4 (C-4″), 69.7 (C-5″), and 68.5 (C-6″), 17.9 (C-6‴). These data are consistent with the published data for rutin [19].
Fr4-2: myricetin-3′-α-L-rhamnopyranoside, yellow powder, 377.2 mg, ESI-MS *m*/*z* 463.26 [M-H]^−^, calc. for C_21_H_20_O_12_, *m*/*z* 464.10). ^1^H NMR (600 MHz, DMSO-*d*_6_): 7.47 (1H, d, *J* = 2.0 Hz, H-2′), 7.46 (1H, d, *J* = 2.0 Hz, H-6′), 6.37 (1H, d, *J* = 2.0 Hz, H-8), 6.18 (1H, d, *J* = 2.0 Hz, H-6), 5.22 (1H, d, *J* = 1.3 Hz, H-1″), 3.77~3.28 (4H, m, sugar protons), and 1.16 (3H, d, *J* = 6.2 Hz, H-6″). ^13^C NMR (151 MHz, DMSO-*d*_6_): 175.9 (C-4), 163.9 (C-7), 160.8 (C-5), 156.1 (C-9), 146.4 (C-2), 146.0 (C-3′), 144.7 (C-5′), 138.6 (C-4′), 136.0 (C-3), 120.8 (C-1′), 110.1 (C-6′), 109.8 (C-2′), 103.0 (C-10), 100.3 (C-1″), 98.2 (C-6), 93.2 (C-8), 72.0 (C-4″), 70.3 (C-3″), 70.1 (C-2″), 69.5 (C-5″), and 17.9 (C-6″). The data are in agreement with those on myricetin-3′-α-L-rhamnopyranoside [20,21].

**Figure 6:**
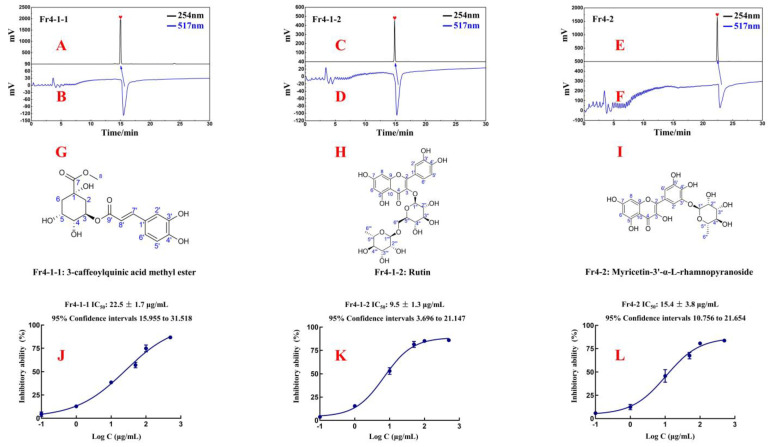
Purity and free-radical-inhibiting activity of Fr4-1-1 (**A**,**B**), Fr4-1-2 (**C**,**D**), and Fr4-2 (**E**,**F**) in ReproSil-Pur C18 AQ analytical column. Chemical structures of Fr4-1-1 (**G**), Fr4-1-2 (**H**), and Fr4-2 (**I**). DPPH inhibitory activities and IC_50_ values of Fr4-1-1 (**J**), Fr4-1-2 (**K**), and Fr4-2 (**L**) at different concentrations.

In order to evaluate the DPPH scavenging activities of these isolated compounds, Fr4-1-1, Fr4-1-2, and Fr4-2, a DPPH scavenging assay was used in this paper. As shown in Figure 6J–L, these three isolated antioxidants displayed strong antioxidant activity with IC_50_ values of 22.5 ± 1.7 μg/mL, 9.5 ± 1.3 μg/mL, and 15.4 ± 3.8μg/mL.

### 2.4. Molecular Docking

**Figure 7:**
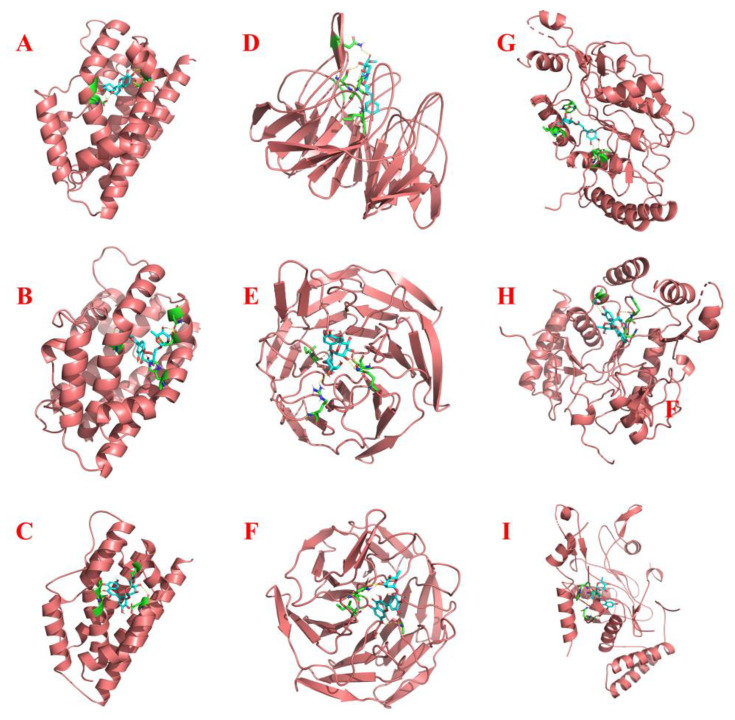
Three-dimensional interaction diagram between HO-1 and Fr4-1-1 (**A**). Three-dimensional interaction diagram between HO-1 and Fr4-1-2 (**B**). Three-dimensional interaction diagram between HO-1 and Fr4-2 (**C**). Three-dimensional interaction diagram between Nrf2 and Fr4-1-1 (**D**). Three-dimensional interaction diagram between Nrf2 and Fr4-1-2 (**E**). Three-dimensional interaction diagram between Nrf2 and Fr4-2 (**F**). Three-dimensional interaction diagram between iNOS and Fr4-1-1 (**G**). Three-dimensional interaction diagram between iNOS and Fr4-1-2 (**H**). Three-dimensional interaction diagram between iNOS and Fr4-2 (**I**).

The HO-1, Nrf2, and iNOS proteins were selected for molecular docking with isolated antioxidants in this study. The detailed docking information as well as the final docking results are displayed in Table 1 and Figure S14. Figure 7 illustrates the steric docking poses of each protein with the antioxidants exhibiting the lowest binding energy, and the specific docking site 2D interaction diagrams are outlined in Figure S13. Overall, all three antioxidants demonstrated excellent binding abilities to the selected proteins with binding values below −5 kcal/mol, thereby reinforcing the significance of our work. Meanwhile, the results obtained for the docking ligands from the target proteins’ original files with the respective proteins also indicated the reliability of AutoDock (Figures S14 and S15). All three antioxidants showed better docking scores than the positive control (inhibitor–protein docking) when docked with HO-1 and iNOS.

Specifically, regarding HO-1, within the active structural domain constituted by Gly-143, Gly-139, Gly-144, Asp-140, Met-34, Glu-29, and Gln-38, Fr4-2 assumed the optimal conformation in its interaction with this protein (Figure 7C and Figure S13C). For Nrf2, Fr4-1-1 exhibited a stronger binding affinity with it, primarily interacting via hydrogen bonds with the amino acid residues Ser-363, Asn-382, Asn-414, Gly-364, Ile-416, and Val-463 (Figure 7D and Figure S13D). As for iNOS, the cavity constructed by Tyr-483, Ser-236, Cys-194, Ile-195, and Trp-366 showed the strongest docking score with Fr4-2 (Figure 7I and Figure S13I).

## 3. Discussion

While traditional Chinese medicine has gained increasing global recognition in recent years, it is imperative to point out that ambiguous bioactive compositions remain a significant barrier to the further development of traditional Chinese medicine [22,23]. With a wide range of Chinese medicinal materials, rich secondary metabolites have always been an important source for lead compound libraries. The comprehensive characterization of bioactive components in Chinese medicinal materials via modern spectroscopic techniques, as well as the exploration of structure–activity relationships, hold profound significance for the in-depth development of traditional Chinese medicine [24]. *R. himalense* is predominantly found in the Asian region at altitudes ranging from 1200 to 4000 m. In the Tibetan and Uighur ethnic communities of China, *R. himalense* has been used for nearly a thousand years in the treatment of vascular diseases, hepatitis, oxidative stress, and other ailments. Despite the excellent bioactivity of *R. himalense*, the lack of a clear pharmacological basis caused by the past empirical drug strategy has hindered the modern pharmaceutical application of this medicinal herb. Based on the previously established chromatographic separation system, our group speculated that the main fractions of *R. himalense* are flavonoids. However, remarkable progress is yet to be made in the study of trace fractions. In this paper, in order to ensure the excellent reproducibility of crude sample pretreatment, the ethanol extract of *R. himalense* was concentrated to 0.5 L and directly mixed with 105 g of polyamide for drying. An additional small chromatographic tower (Figure S1A) was used to load the sample to protect the medium-pressure chromatographic column and improve reproducibility. After 13 cycles were performed, Figure 1B–F reveals that there was almost no crossover between the recovered fractions following the sample pretreatment, covering all visual peaks in the crude sample. The presence of Fr1 as well as Fr4 confirmed the excellent ability of polyamide-MPLC to enrich trace compounds.

The continuous introduction of novel modified filler chromatographic columns has improved the selectivity for complex compounds [25,26]. In contrast with conventional octadecylsilica (ODS) columns, phenyl columns contain phenyl functional group π-electrons, which can provide additional hydrogen bonding, π–π interactions, and ionic interactions [27]. These properties allow the combination of phenyl columns and ODS columns to enhance the potential to separate complex compounds. Without considering interference from other conditions, the chromatographic peak of the compound was expected to follow a Gaussian distribution; however, it was observed that Fr4-1 did not exhibit a satisfactory performance in the Reprosil-Pur C18 AQ analytical column. Consequently, we employed a Gaussian function to fit the chromatographic peak, and the calculated half-peak width was determined to be 2.6. Visual inspection of Figure 3A revealed significant disparities between the fitted chromatographic peak and actual data, indicating poor resolution. In addition, the tailing factor of this peak was also calculated to be 1.84, which suggested that further analysis and purification of this component was required. Subsequent chromatographic analysis further corroborated this observation, whereby Fr4-1 showed two visually separable peaks in the Kromasil 100-5-phenyl analytical column (Figure 4B). Based on this, the Kromasil 100-5-phenyl column was selected for further processing of Fr4-1. Upon examining the structures of rutin and 3-caffeoylquinic acid methyl ester, we hypothesized that a difference in aromaticity supported the above experimental results.

Oxidative stress, as a normal physiological immune response in the body, is closely associated with the pathogenesis of many diseases [28]. Therefore, in modern pharmacological research, antioxidants have been widely explored not only in regulating oxidative imbalance but also in the treatment of various diseases [29,30]. As important sources of exogenous antioxidants, the isolation and activity investigation of flavonoids and phenylpropanoids have received much attention from the scientific community [31,32]. In this paper, bioactivity assay and targeted separation were integrated into a series, in which the online HPLC-DPPH was responsible for the activity determination of the sample, while MPLC and HPLC were responsible for the targeted separation of antioxidants. Finally, 3-caffeoylquinic acid methyl ester, rutin, and myricetin-3′-α-l-rhamnoside were isolated from *R. himalense*. Distinguished from some other methods reported in the literature [33,34,35], our work was entirely realized at the chromatographic level, minimizing the loss of active components and being more environmentally friendly. As far as *R. himalense* is concerned, this marks the first report of these three antioxidants within this plant, which will undoubtedly contribute to the establishment of a bioactive compound library in *R. himalense* and provide a more comprehensive understanding of its medicinal value.

In addition, to further explore the activity of 3-caffeoylquinic acid methyl ester, rutin, and myricetin-3′-α-L-rhamnopyranoside, computer-based virtual docking was incorporated into this study. AutoDock is an open-source software developed by the Center for Computational Structural Biology specifically applied to ligand–protein docking, and its excellent performance has been confirmed by several studies [36,37]. The protein structure downloaded from the PDB database contained small-molecule ligands that have been shown to bind stably. We separated the protein and ligand for re-docking. Finally, each protein and its original ligand showed a strong binding ability, which also confirmed the reliability of AutoDock. In order to further evaluate the activity of three antioxidants, we searched the internet for small-molecule inhibitors of selected proteins and performed docking (positive control). Compared with positive controls, the antioxidants isolated in this study primarily interacted with target proteins via hydrogen bonding, which is likely attributed to their abundant hydroxyl groups. In contrast, inhibitors of these target proteins not only established connections via hydrogen bonding but also involved a multitude of interactions such as amide-pi stacked, Pi-Alkyl, Pi-Sulfur, and other interactions. The experimental results imply that these compounds have the ability to autonomously establish a stable binding state with potential target proteins via the active sites, influencing downstream signaling pathways and exerting their physiological functions. However, considering the crucial role of docking proteins in inflammatory diseases, tumors, and cardiovascular diseases [38,39,40], it is necessary to further explore the roles played by these three antioxidants in disease control via more rigorous pharmacological experiments in subsequent studies.

In summary, this study utilized chromatographic technology to efficiently identify and separate free radical inhibitors in *R. himalense* and further elucidate the pharmacologically active constituents of this plant, as well as its significant medicinal value. The isolated antioxidants were then virtually docked with target proteins, which provided research ideas for the subsequent pharmacological experiments.

## 4. Materials and Methods

### 4.1. Chemicals and Reagents

The HR-ESI-MS, ESI-MS, and NMR spectra were documented using an Agilent 6500 Q-TOF mass spectrometer (Agilent, Santa Clara, CA, USA), a Waters ZQ 2000 mass spectrometer (Waters, Milford, MA, USA), and a Bruker Avance 600 MHz (Bruker, Karlsruhe, Germany), respectively. Two instruments, an LC-10AD and LC-16, were utilized for the online analysis of HPLC-DPPH activity. Each was coupled to a Shimadzu workstation (Shimadzu, Kyoto, Japan), a UV–Vis detector, and two binary gradient pumps. Furthermore, these instruments were integrated using triple valves and polyetheretherketone reaction coils measuring 18.0 m by 0.25 mm in diameter (the scheme is shown in Figure 2A). A preparative HPLC system (Hanbon, Huai’an, China) with a polyamide (100–200 mesh) medium-pressure column (49 × 460 mm) was set up in order to process the crude samples. The information on the other chromatographic columns was as follows: Kromasil 100-5-Phenyl analytical and preparative columns (4.6 × 250 mm and 20 × 250 mm, 5 μm; Kromasil, Gothenburg, Sweden) and two Reprosil-Pur C18 AQ columns (4.6 × 250 mm and 20 × 250 mm, 5 μm; Maisch, Munich, Germany). DPPH was acquired from Sigma-Aldrich (Steinheim, Germany). HPLC-grade methanol and acetonitrile (ACN), as well as analytical-grade methanol and ethanol, were used. These reagents were acquired from Xinlanjing Chemical Industry (Yuxi, China) and the Kelon Chemical Reagent Factory (Chengdu, China). Utilizing a Moore water purification system, HPLC-grade H_2_O was produced (Chongqing, China).

### 4.2. Sample Extraction, Polyamide-MPLC Pretreatment, and Activity Screening

*R. himalense*’s above-ground parts (leaves and stems) were gathered from Xiewu Town in the Yushu Tibetan Autonomous Prefecture of Qinghai Province, China (95°05′31″ E, 33°08′45″ N), and they were authenticated by Prof. Lijuan Mei of the Northwest Institute of Plateau Biology, the Chinese Academy of Sciences. The Qinghai-Tibetan Platea Museum of Biology, the Chinese Academy of Sciences, had a sample (no. 0342650) in its possession.

The schematic diagram of the extraction process of *R. himalense* is shown in Figure S2. Specifically, after air drying and crushing the harvested samples to obtain 300 g of powder, extraction was performed at room temperature for 12 h using 8 L of 95% ethanol, with the extraction process repeated 3 times for a total of 24 L of the solution. This was followed by filtration and concentration in a rotavapor at 40 °C until the volume decreased to 0.5 L. At this point, 105 g of polyacrylamide was added to the 0.5 L concentrated solution, and then the mixture was placed in a drying oven at 60 °C.

An empty chromatographic tower (49 × 100 mm) was filled with the polyamide crude extract (130.3 g in total). A polyamide chromatographic column (49 × 460 mm) and preparative HPLC apparatus were linked to it for pretreatment. ACN and water were utilized as the mobile phase for the sample separation, with a linear elution gradient of 0–100 min, 0–100% ACN, and a flow rate of 57 mL/min. Throughout the procedure, the absorbance at 254 nm was monitored, and after 13 separations, 4 fractions (labeled Fr1, Fr2, Fr3, and Fr4) were eventually attained.

The effect of the polyamide-MPLC pretreatment was studied by analyzing the crude sample, along with the 4 recovered fractions, under the same chromatographic conditions. In this case, a Reprosil-Pur C18 AQ analytical column was utilized, and the concentration and injection volume were the same for each sample to be analyzed (4 mg/mL; 5 μL). ACN and water were employed as the mobile phase, and the linear elution gradient was 0–60 min with 5–95% ACN. At 254 nm, chromatographic measurements were recorded.

The target fraction Fr4 was solubilized in 8.0 mL of methanol, and the resulting mixture was processed through an organic membrane (0.45 μm) before its activity was assessed and additional separation was carried out. Then, a Reprosil-Pur C18 AQ analytical column was used in the online HPLC-DPPH activity screening process to recognize free radical inhibitors in Fr4. In this case, the mobile phase contained ACN and water, flowing at a rate of 1 mL/min, with the isocratic elution gradient as follows: 0 to 50 min, and 5% to 55% ACN. In addition, the DPPH had a concentration of 50 μg/mL and a flow rate of 0.8 mL/min, whereas the chromatographic data and findings of free radical scavenging activity were acquired at 254 nm and 517nm.

### 4.3. Directed Separation of Antioxidants from Fr4

Employing a Reprosil-Pur C18 AQ preparative column with a mobile phase comprising water and ACN flowing at a constant rate of 19 mL/min, Fr4 was further separated. An elution gradient, whereby the ACN content was linearly increased from 5% to 55% over 50 min, was applied. As before, chromatograms were acquired at 254 nm.

The above separation process yielded 2 main fractions (labeled Fr4-1 and Fr4-2) after dissolving the former (Fr4-1; 104.2 mg) in 1.0 mL methanol. Using a 5–55% ACN gradient, analysis was performed in a Reprosil-Pur C18 AQ analytical column for 50 min. In addition, this fraction was eluted in a Kromasil 100-5-Phenyl analytical column with a 5–95% ACN gradient for 50 min, with all chromatographic results obtained at 254 nm.

After analyzing Fr4-1 with 2 chromatographic columns, it was finally decided to utilize a Kromasil 100-5-Phenyl preparative column for the sample separation. For this purpose, an isocratic elution gradient was also applied for 40 min at an ACN concentration of 11%, whereas the mobile phase consisted of water and ACN at a constant flow rate of 19 mL/min. As before, the elution of fractions was monitored at 254 nm.

### 4.4. Activity and Purity of Antioxidants

The separated molecules’ purity and free radical scavenging capacity were assessed via the online HPLC-DPPH activity screening. In this case, a linear elution gradient consisting of ACN and water as the mobile phases was applied as follows: 5–55% ACN for 30 min at 1 mL/min. DPPH was used at a concentration of 50 μg/mL. While monitoring at 517 nm, the free radical scavenging activity was eventually found. Based on the literature [41], a slightly modified DPPH scavenging assay method was used to evaluate the scavenging ability of the free radical inhibitors. For this purpose, before adding 30 μL of each sample to 70 μL of DPPH (0.25 mg/mL) solution in a 96-well microtiter plate, sample solutions of varying concentrations (0.1, 1, 10, 50, 100, and 500 μg/mL) were prepared. After 30 min of dark incubation, the mixtures’ absorbance readings were recorded at 517 nm. From the results, the antioxidant activities of molecules were measured as follows:

Inhibitory activity (%) = [1 − (A1 − A2)/A3] × 100%

A1 represents the absorbance of the sample solution, A2 represents the absorbance of the blank, and A3 represents the absorbance of the control, respectively.

### 4.5. Computer Simulation

The 3D structures of the small-molecule compounds were drawn using ChemBio3D software (version: 16.0.1.4) and exported as a mol2 file. The mol2 files of inhibitors of HO-1 (Heme Oxygenase-1-IN-1, CAS: 1093058-52-6), Nrf2 (ML385, CAS: 846557-71-9), and iNOS (Isoquercetin, CAS: 482-35-9) were also obtained using the above methods. The crystal structures of target proteins were retrieved and downloaded from the online RCSB protein database (https://www.rcsb.org/, accessed on 15 August 2023), as follows: heme oxygenase-1 (HO-1, PDB ID: 1N3U), nuclear factor erythroid 2-related factor 2 (Nrf2, PDB ID: 4IQK), and inducible nitric oxide synthase (iNOS, PDB ID: 2ORO). The open-source software PyMOL and AutoDock4 were utilized to preprocess these molecules for docking, which included dehydration, ligand removal, and charge addition. The PDB file of the original ligand of each target protein was also obtained using PyMOL. Subsequently, the active pocket coordinates of receptor proteins were created using the AutoGrid module. Docking was performed for 100 iterations, with the maximum number of evals set to “long”, and the other settings were maintained at their default values. Finally, PyMOL (version: 2.5) and LigPlus software (version: 2.2) were employed for the analysis and visualization of the docking results with the lowest binding energy.

### 4.6. Statistical Analysis

Using SPSS 20.0 (SPSS, Chicago, IL, USA), the data from the three replicated tests were examined, and the results were presented as means and standard deviation. The concentrations of the separated free radical inhibitors that induced 50% inhibition (IC_50_) of the scavenging activity were determined using nonlinear regression. The curves for the scavenging activities of DPPH were eventually created with Prism 8.0 software.

## 5. Conclusions

Our aim was to develop more strategies for investigating active compounds in natural products and enriching the lead compound library. This study combined polyamide-MPLC, an online HPLC-DPPH activity screening system, and HPLC to efficiently identify and separate antioxidants in *R. himalense* trace fractions. The isolated compounds, 3-caffeoylquinic acid methyl ester, rutin, and myricetin-3′-α-l-rhamnoside, were further subjected to molecular docking with the target proteins of interest, providing an in-depth exploration of their biological activities from a computational theoretical perspective. In conclusion, the integrated approach established in this study specifically investigated antioxidants in *R. himalense*, offering novel insights and methodological references for the prospective development of natural products as new drugs.

## Figures and Tables

**Table 1:** Molecular docking parameters and results.

Protein	Ligand	Number of Points	Center Grid Box	Spacing	Docking Score
HO-1 ID: 1N3U	Fr4-1-1	X_dimension = 46	X_center = 13.356	0.403	−6.01 kcal/mol
	Fr4-1-2	Y_dimension = 40	Y_center = 1.105		−6.68 kcal/mol
	Fr4-2	Z_dimension = 36	Z_center = 0.763		−6.94 kcal/mol
Nrf2 ID: 4IQK	Fr4-1-1	X_dimension = 34	X_center = −45.033	0.375	−7.01 kcal/mol
	Fr4-1-2	Y_dimension = 56	Y_center = 2.477		−5.73 kcal/mol
	Fr4-2	Z_dimension = 50	Z_center = −15.44		−6 kcal/mol
iNOS ID:2ORO	Fr4-1-1	X_dimension = 40	X_center = 64.041	0.375	−8.47 kcal/mol
	Fr4-1-2	Y_dimension = 50	Y_center = −13.171		−6.49 kcal/mol
	Fr4-2	Z_dimension = 46	Z_center = 50.069		−8.54 kcal/mol

## Data Availability

Data is contained within the article and Supplementary Material.

## Supplementary Materials

The following supporting information can be downloaded at https://www.mdpi.com/article/10.3390/ijms25010227/s1.
