# Supplementary material for: Separation of Antioxidants from Trace Fraction of Ribes himalense via Chromatographic Strategy and Their Antioxidant Activity Supported with Molecular Simulations
Source: Int J Mol Sci. 2023 Dec 22;25(1):227. doi: 10.3390/ijms25010227 (PMC10778596; doi:10.3390/ijms25010227)

Supporting Information

Separation of antioxidants from trace fraction of *Ribes himalense* via chromatographic strategy and their antioxidant activity supported by molecular simulations

Youyi Liu[1, †], Chuang Liu[1,2, †], Yuqing Lei[1,2], Jingrou Guo[1], Xingyi Chen[1], and Minchen Wu[1, *]

[1]*Wuxi School of Medicine, Jiangnan University, Wuxi 214122, China*

[2]*School of Biotechnology, Jiangnan University, Wuxi 214122, China*

Table of Contents

Figure S1. Apparatus diagrams 3

Figure S2. Schematic diagram of substance extraction process 4

Figure S3. ESI-MS data of Fr4-1-1 5

Figure S4. ^{1}H NMR (600 MHz) data of Fr4-1-1 (in DMSO-d_6) 6

Figure S5. ^{13}C NMR (151 MHz) data of Fr4-1-1 (in DMSO-d_6) 7

Figure S6. HR-ESI-MS data of Fr4-1-2 8

Figure S7. ^{1}H NMR (600 MHz) data of Fr4-1-2 (in MeOH-d_4) 9

Figure S8. ^{13}C NMR (151 MHz) data of Fr4-1-2 (in MeOH-d_4) 10

Figure S9. HMBC data of Fr4-1-2 (in MeOH-d_4) 11

Figure S10. ESI-MS data of Fr4-2 12

Figure S11. ^{1}H NMR (600 MHz) data of Fr4-2 (in DMSO-d_6) 13

Figure S12. ^{13}C NMR (151 MHz) data of Fr4-2 (in DMSO-d_6) 14

Figure S13. Docking results of antioxidants with target proteins 15

Figure S14. Histogram of all docking results 16

Figure S15. The binding sites of small molecule ligands and target proteins 17

Figure S1. Apparatus diagrams

The actual polyamide-MPLC system (A) and the schematic diagram of online HPLC-DPPH screening system (B).

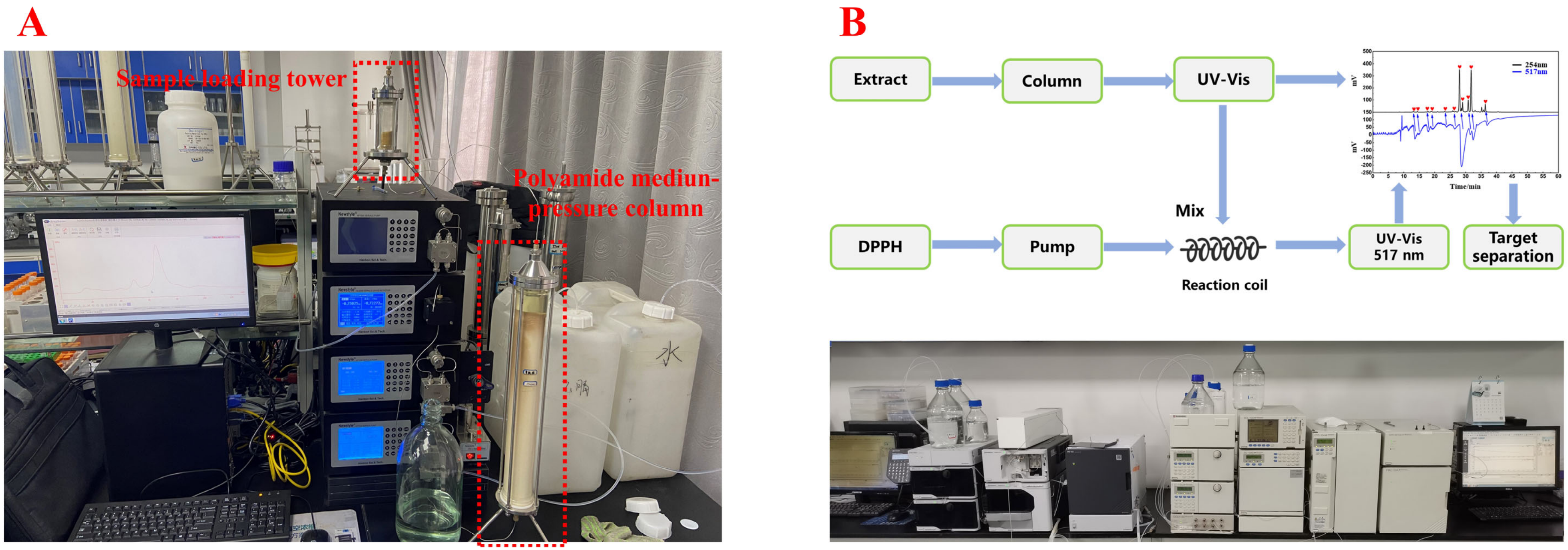

Figure S2. Schematic diagram of substance extraction process

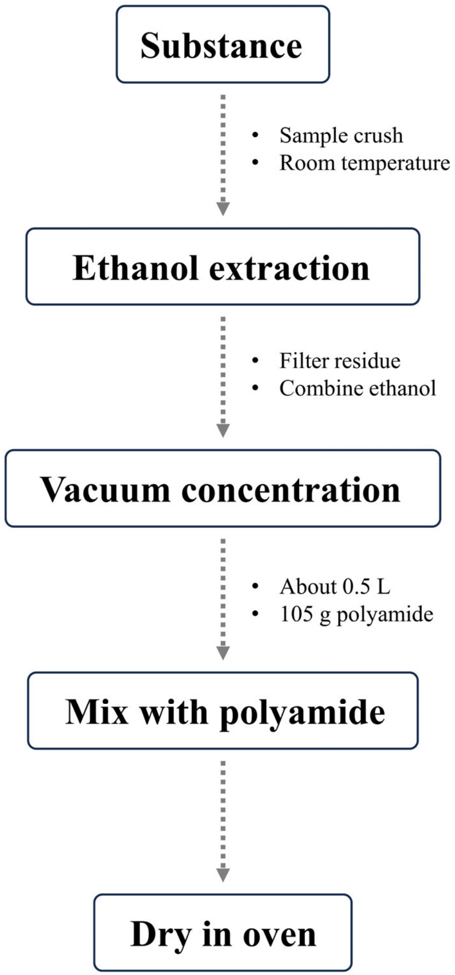

Figure S3. ESI-MS data of Fr4-1-1

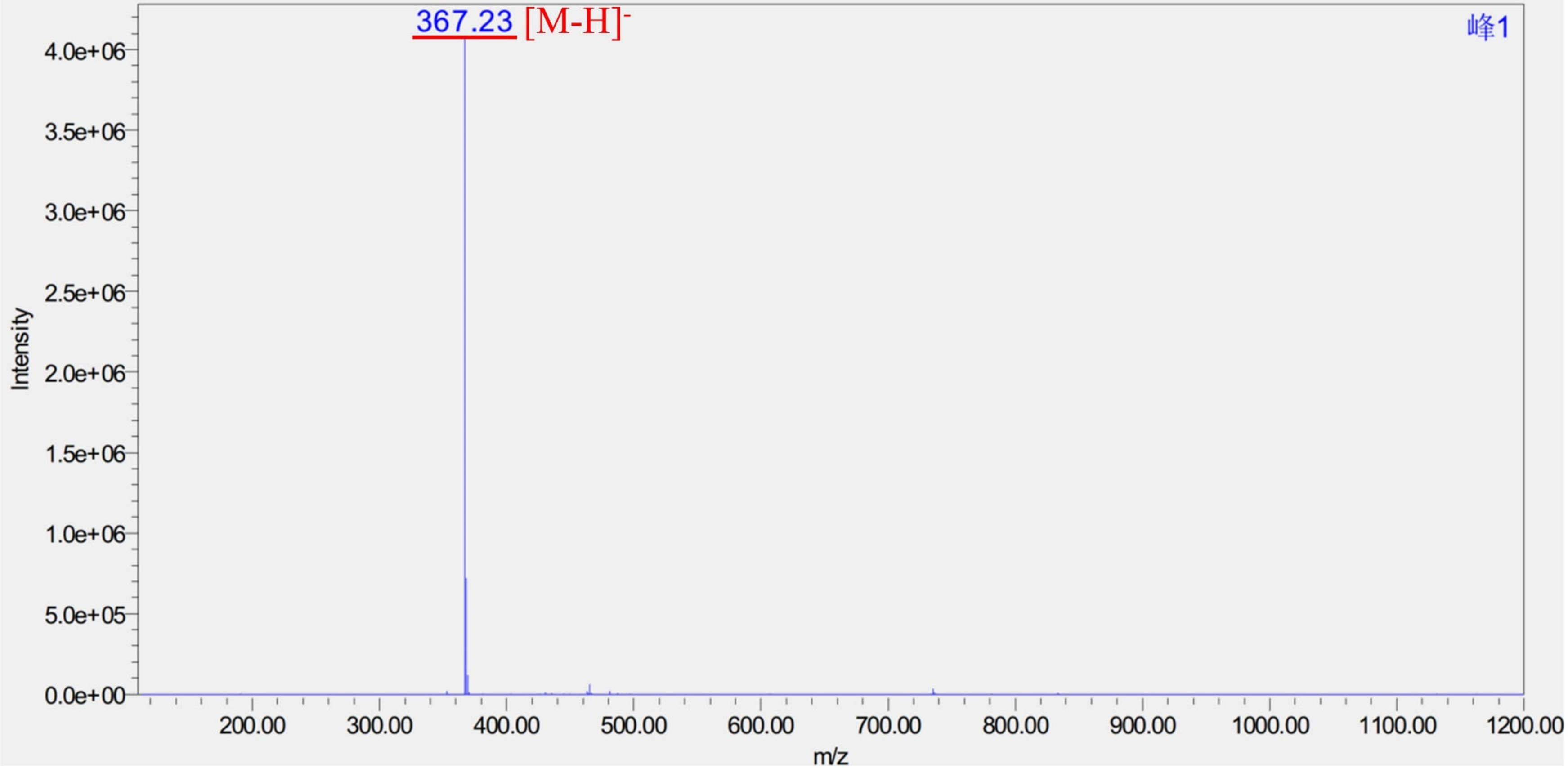

Figure S4. ^{1}H NMR (600 MHz) data of Fr4-1-1 (in DMSO-d_6)

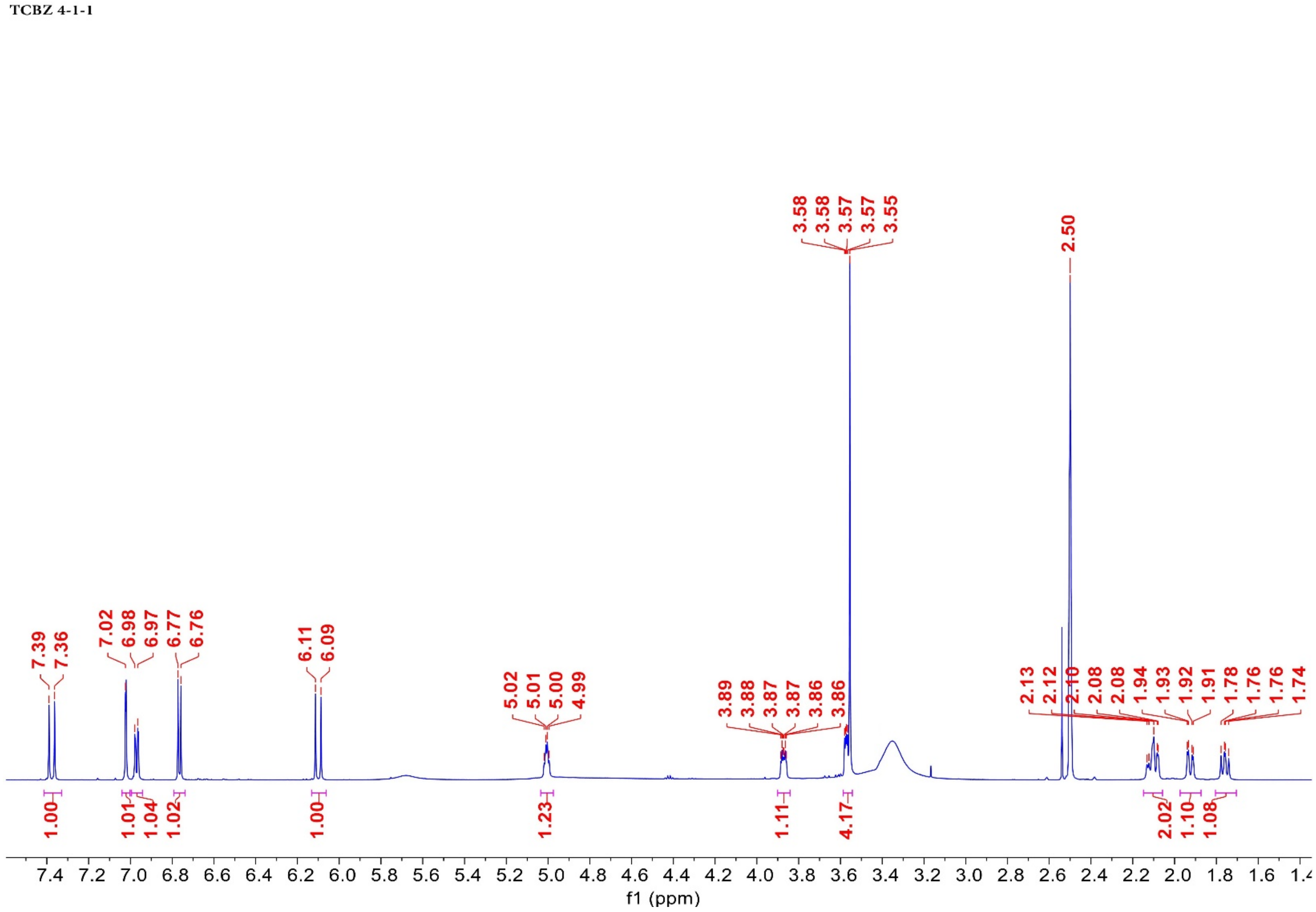

Figure S5. ^{13}C NMR (151 MHz) data of Fr4-1-1 (in DMSO-d_6)

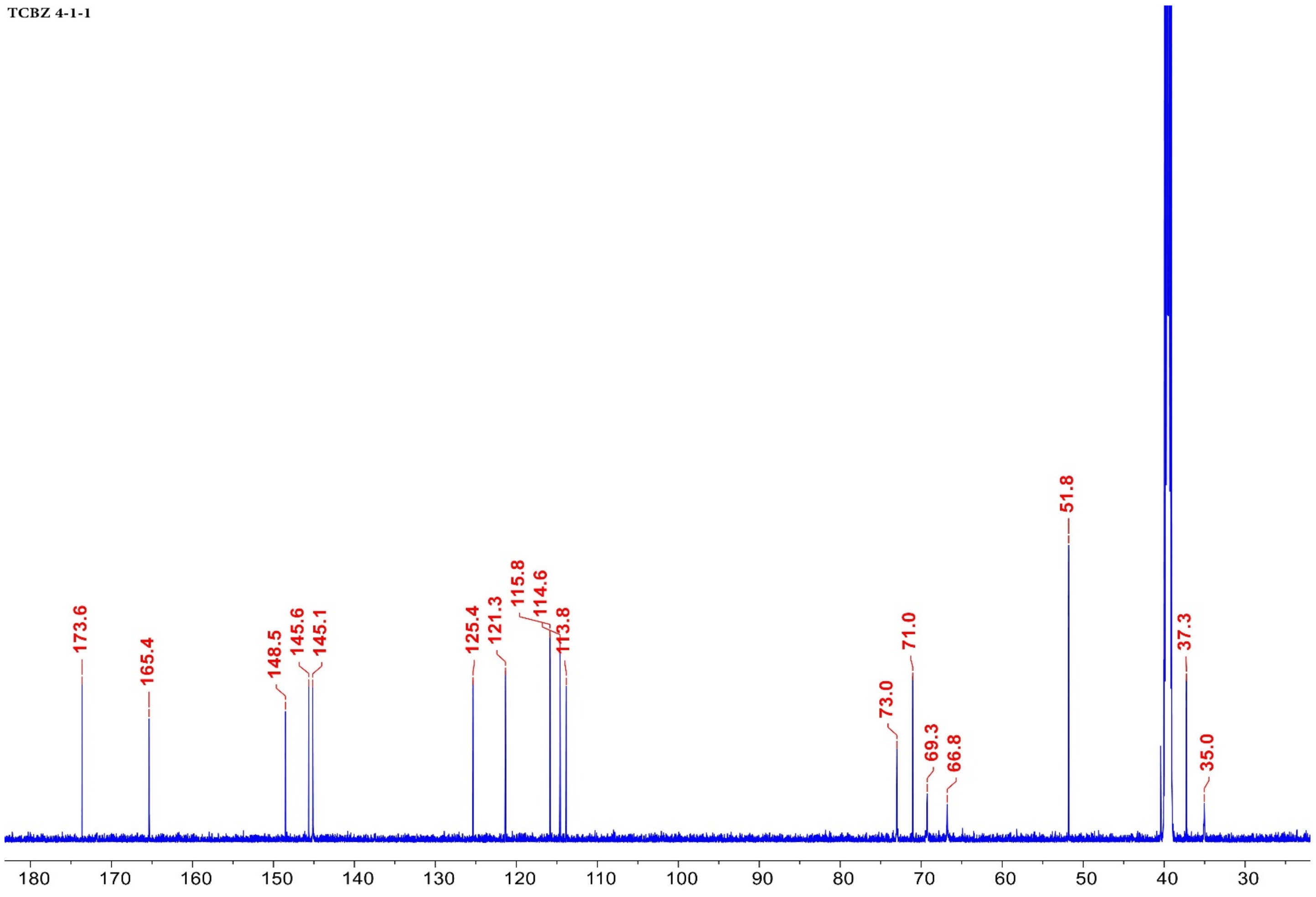

Figure S6. HR-ESI-MS data of Fr4-1-2

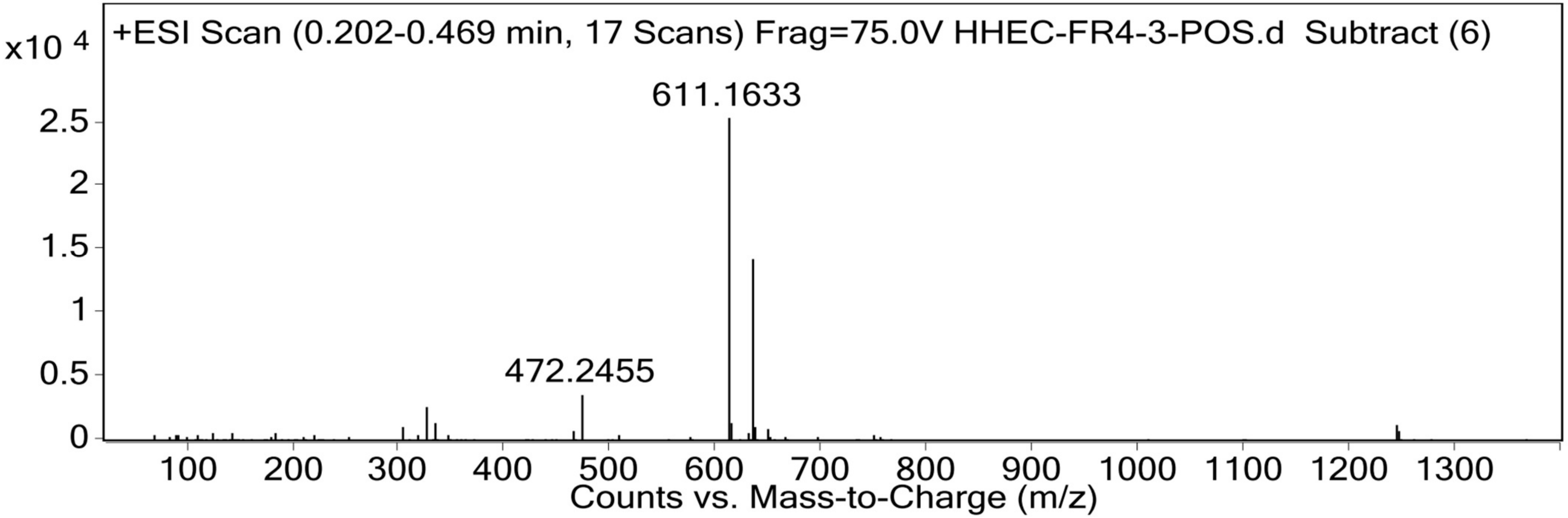

Figure S7. ^{1}H NMR (600 MHz) data of Fr4-1-2 (in MeOH-d_4)

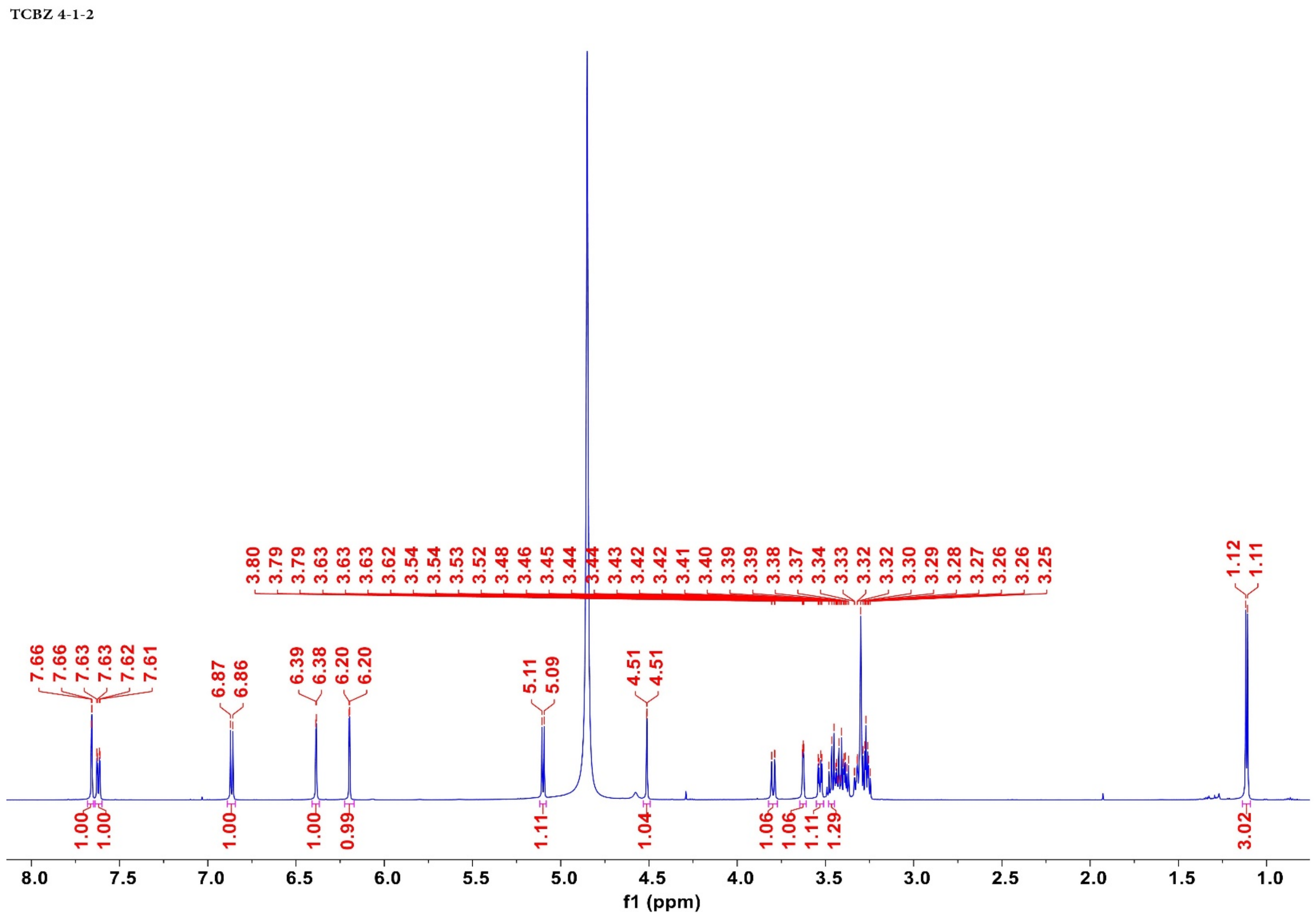

Figure S8. ^{13}C NMR (151 MHz) data of Fr4-1-2 (in MeOH-d_4)

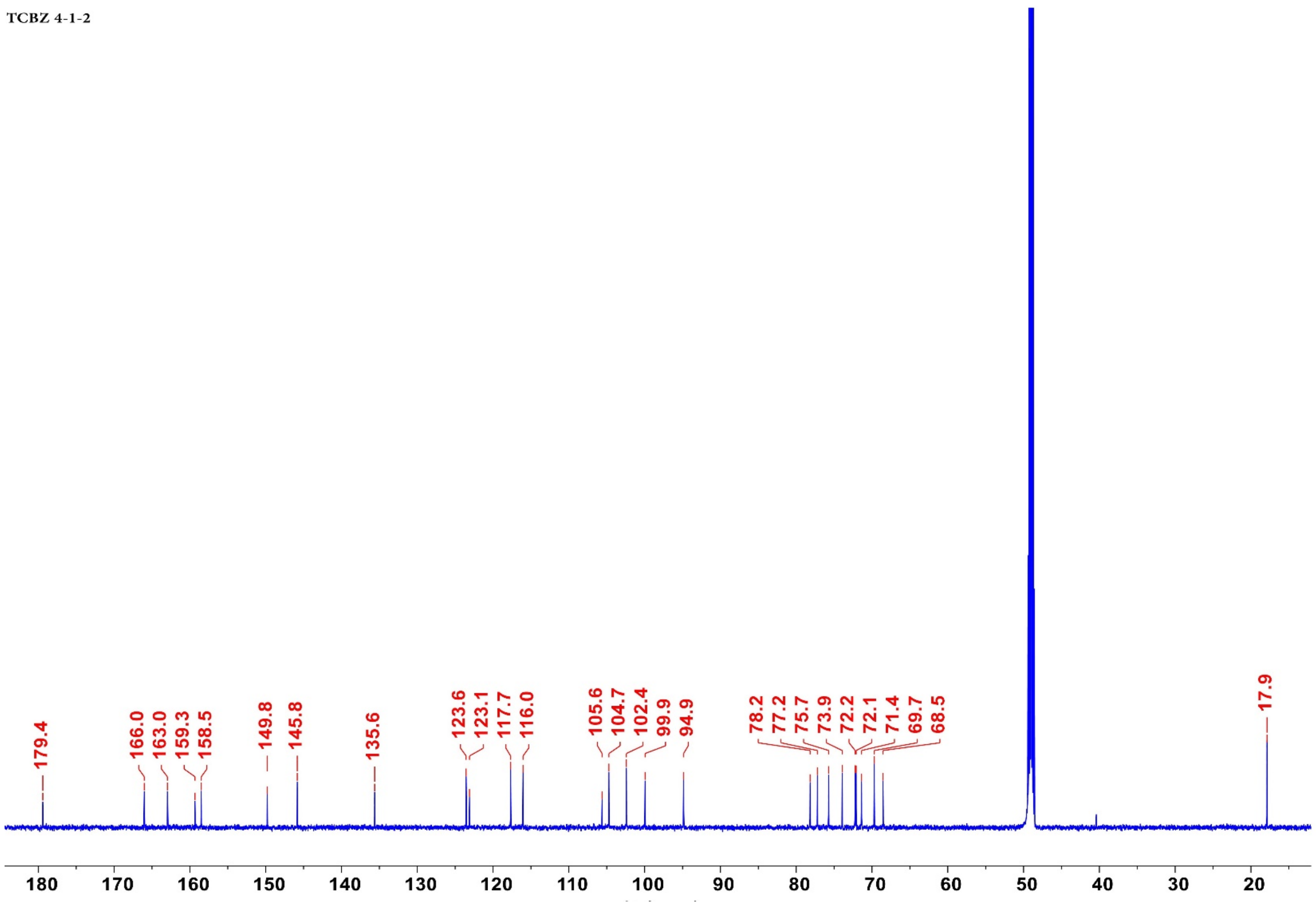

Figure S9. HMBC data of Fr4-1-2 (in MeOH-d_4)

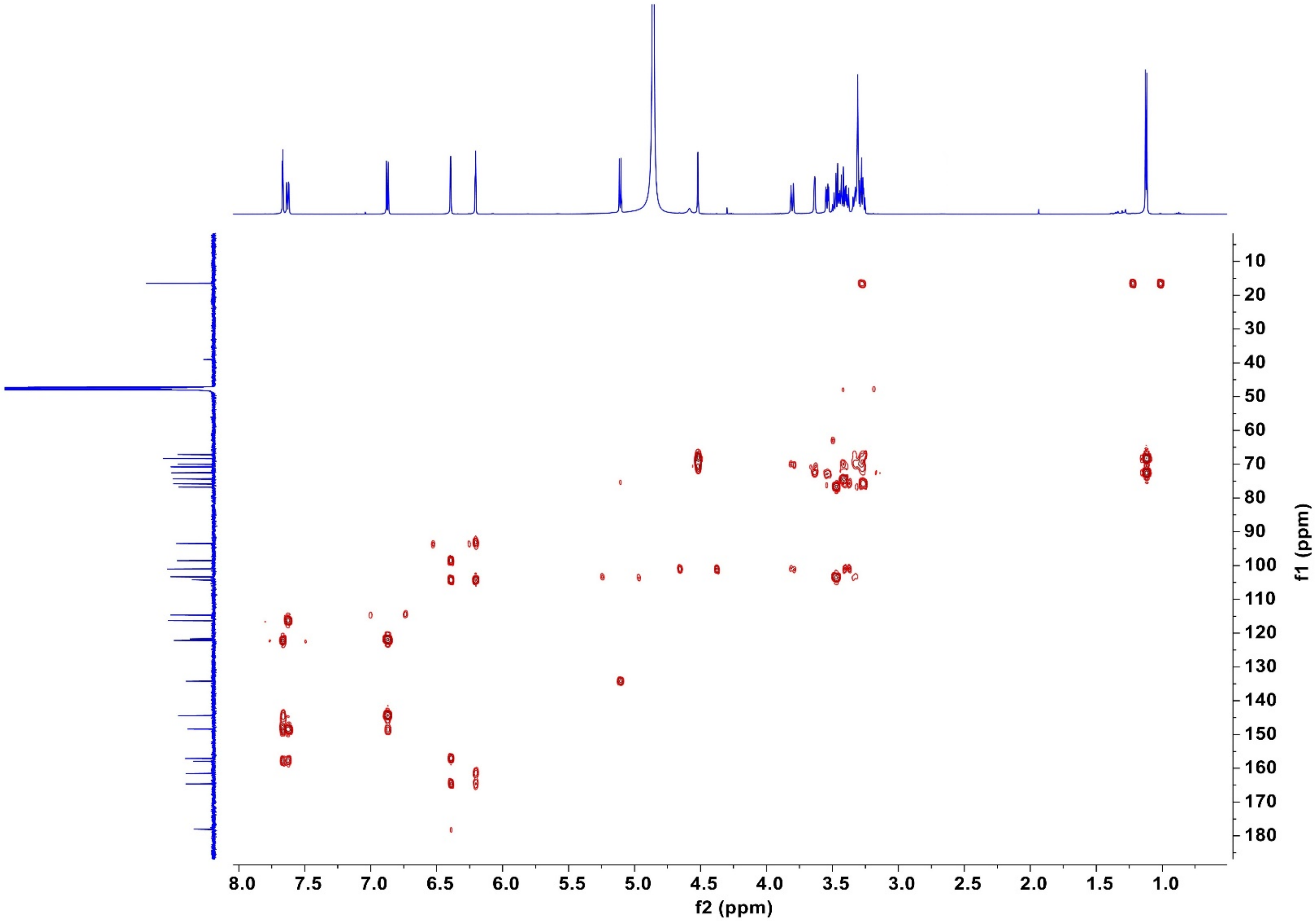

Figure S10. ESI-MS data of Fr4-2

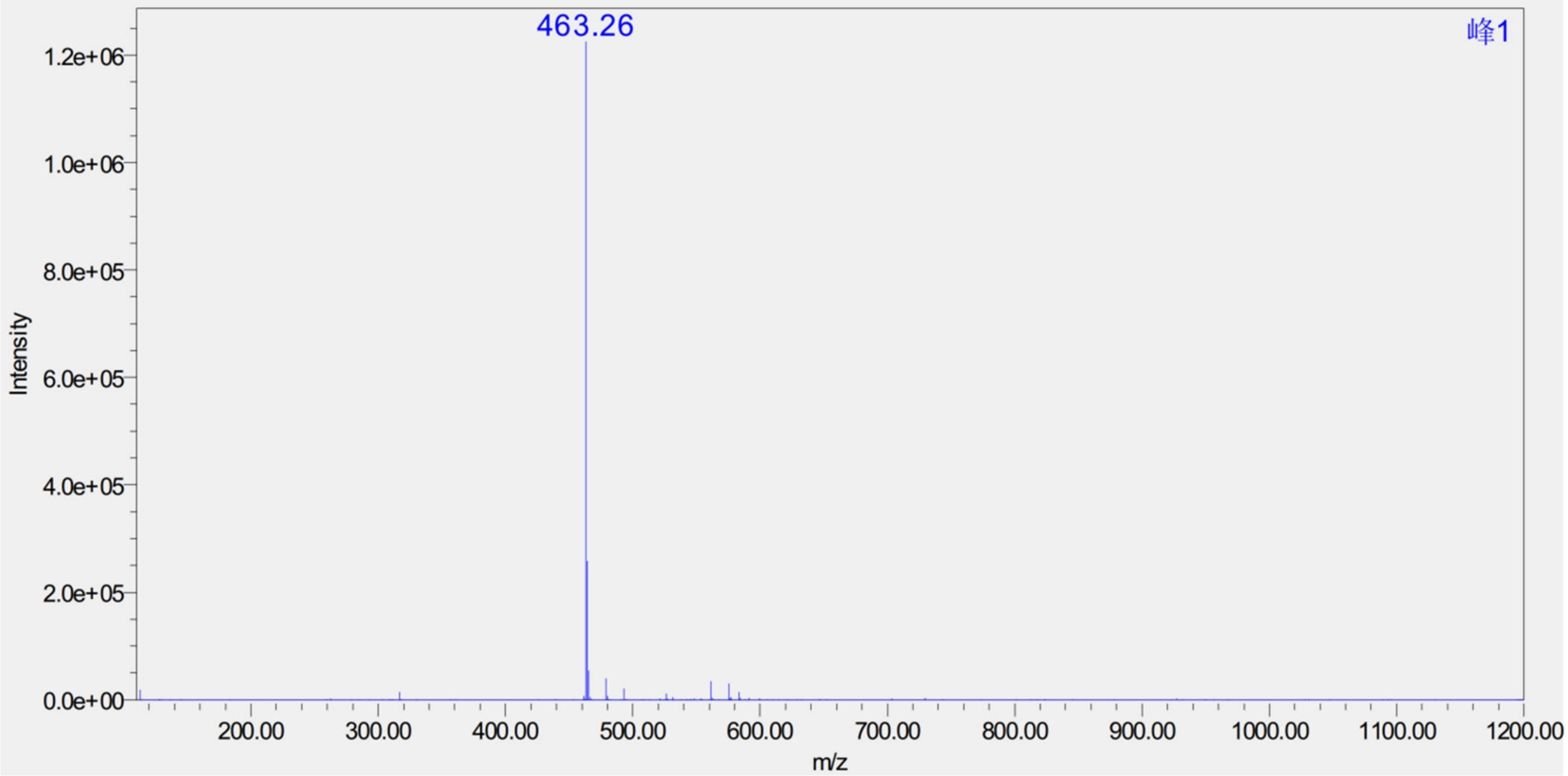

Figure S11. ^{1}H NMR (600 MHz) data of Fr4-2 (in DMSO-d_6)

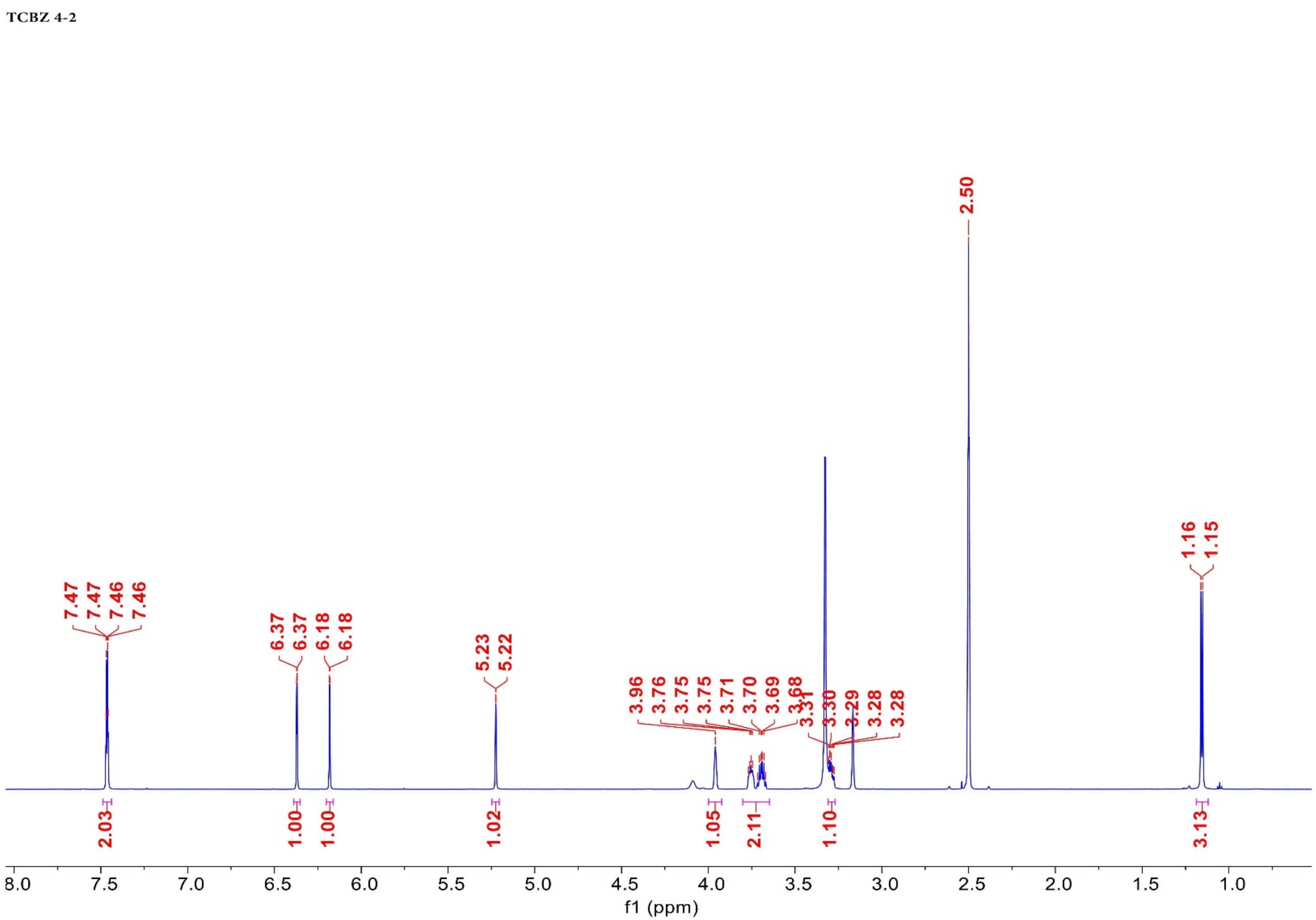

Figure S12. ^{13}C NMR (151 MHz) data of Fr4-2 (in DMSO-d_6)

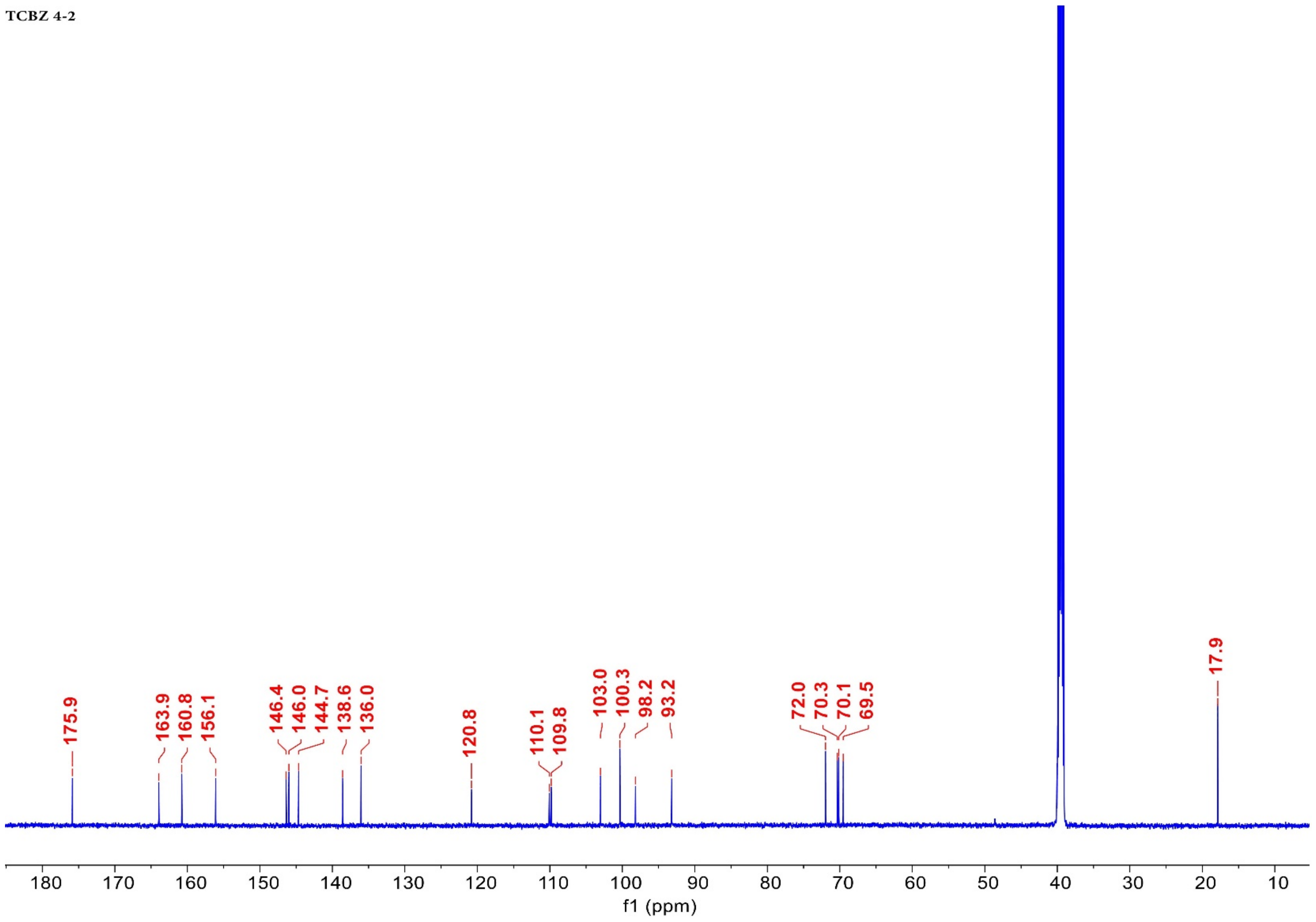

Figure S13. Docking results of antioxidants with target proteins

2D-interaction diagram between HO-1 and Fr4-1-1 (A); 2D-interaction diagram between HO-1 and Fr4-1-2 (B); 2D-interaction diagram between HO-1 and Fr4-2 (C); 2D-interaction diagram between Nrf2 and Fr4-1-1 (D); 2D-interaction diagram between Nrf2 and Fr4-1-2 (E); 2D-interaction diagram between Nrf2 and Fr4-2 (F); 2D-interaction diagram between iNOS and Fr4-1-1 (G); 2D-interaction diagram between iNOS and Fr4-1-2 (H); 2D-interaction diagram between HO-1 and Fr4-2 (I).

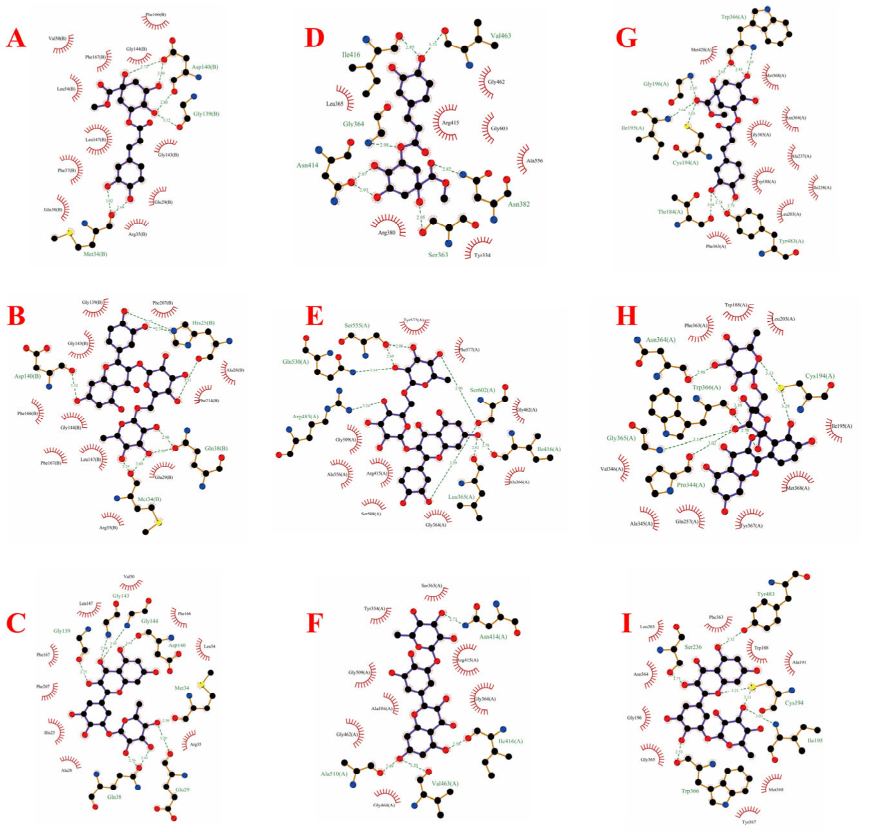

Figure S14. Histogram of all docking results

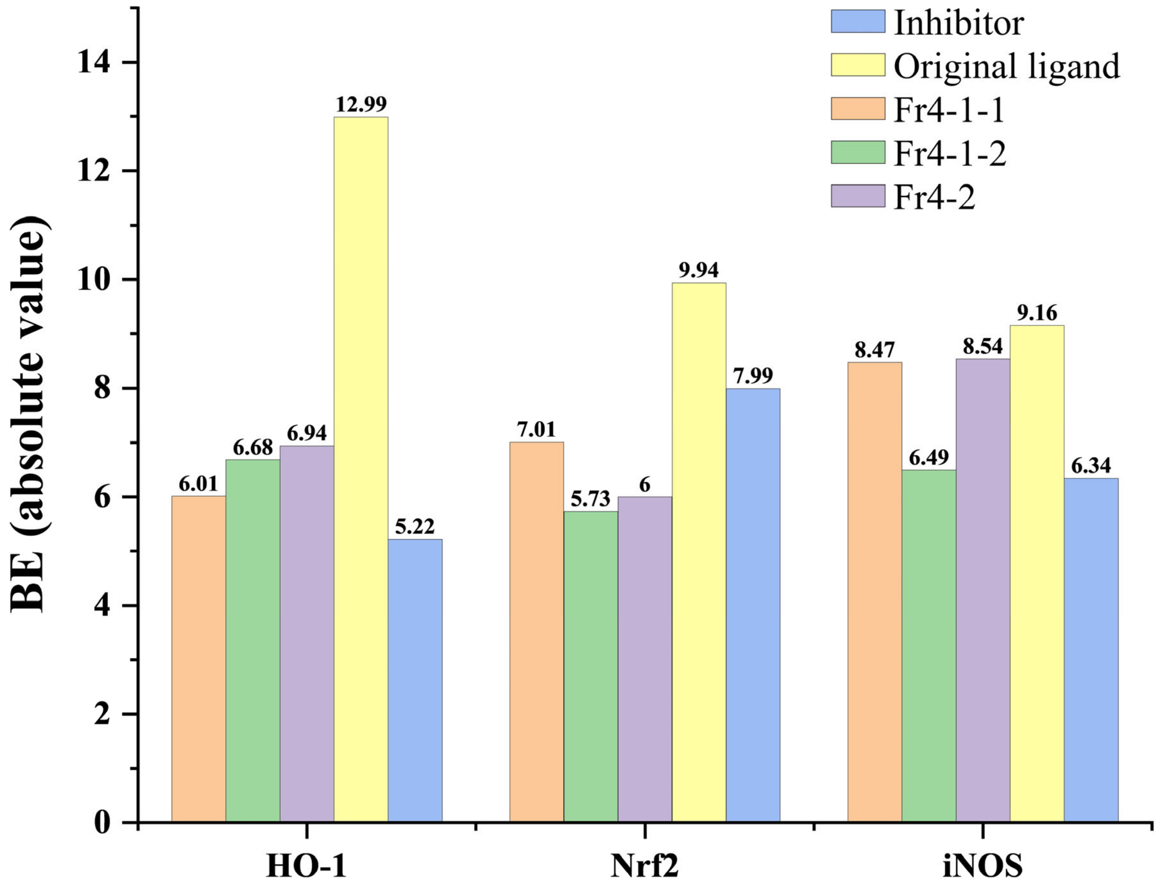

Figure S15. The binding sites of small molecule ligands and target proteins

2D-interaction diagram between HO-1 and Heme Oxygenase-1-IN-1 (A); 2D-interaction diagram between Nrf2 and ML385 (B); 2D-interaction diagram between iNOS and Isoquercetin (C); 2D-interaction diagram between HO-1 and HEM (D); 2D-interaction diagram between Nrf2 and IQK (E); 2D-interaction diagram between iNOS and 228 (F).

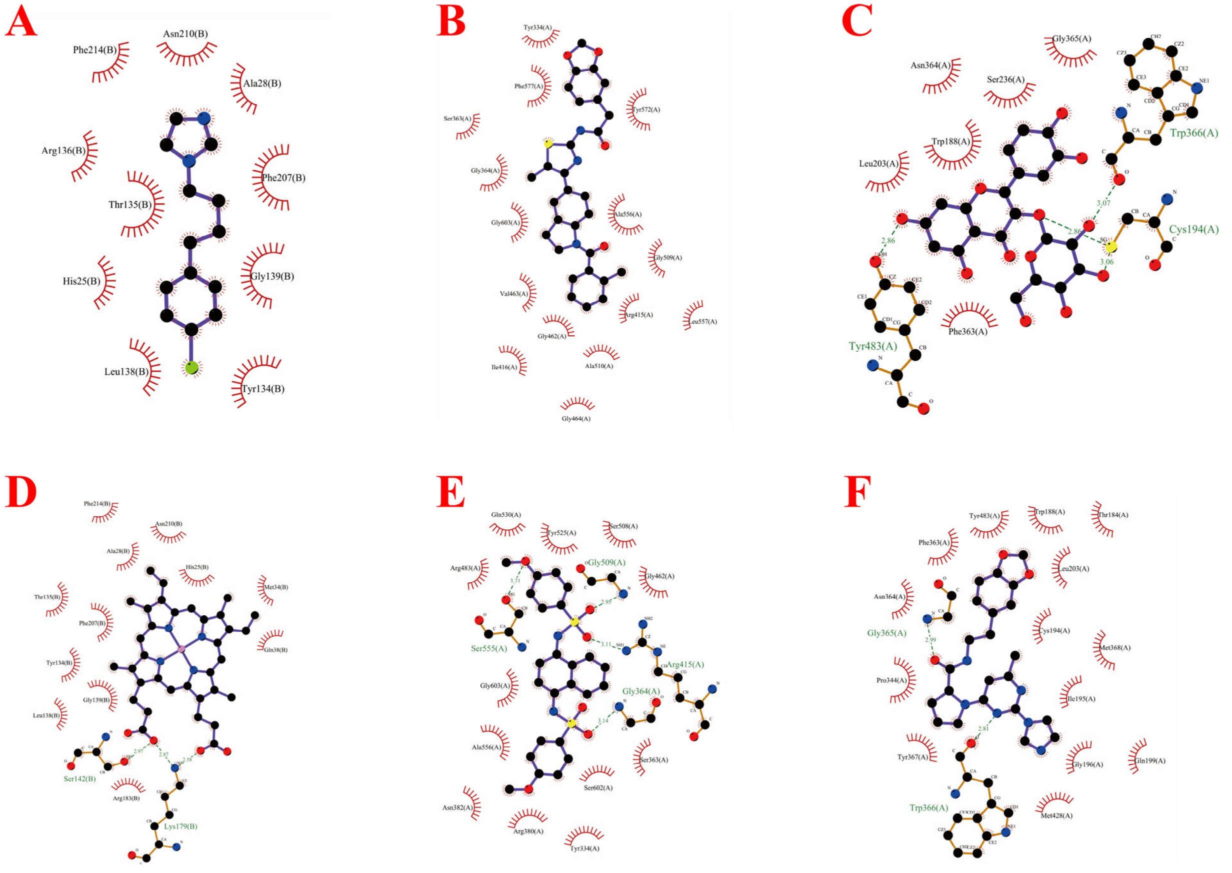

Supplement: Supplementary file 1 [file ijms-25-00227-s001.zip › ijms-2766694-supplementary.pdf]
